# Genetic effects of *Red Lettuce Leaf* genes on red coloration in leaf lettuce under artificial lighting conditions

**DOI:** 10.1002/pei3.10089

**Published:** 2022-09-02

**Authors:** Kaede C. Wada, Noritoshi Inagaki, Hiroaki Sakai, Hiroto Yamashita, Yusuke Nakai, Zui Fujimoto, Jun‐ichi Yonemaru, Hironori Itoh

**Affiliations:** ^1^ Breeding Big Data Management and Utilization Group, Division of Smart Breeding Research, Institute of Crop Science National Agriculture and Food Research Organization Tsukuba Japan; ^2^ Biomacromolecules Research Unit, Research Center for Advanced Analysis, Core Technology Research Headquarters National Agriculture and Food Research Organization Tsukuba Japan; ^3^ Bioinformatics Unit, Research Center for Advanced Analysis, Core Technology Research Headquarters National Agriculture and Food Research Organization Tsukuba Japan; ^4^ Greenhouse Vegetable Production Group, Division of Field Crop and Vegetable Research, Kyushu‐Okinawa Agricultural Research Center National Agriculture and Food Research Organization Kurume Japan

**Keywords:** anthocyanin biosynthesis, artificial light type plant factories, genotyping, leaf lettuce (*Lactuca sativa*), MBW complex, RNA‐seq, transcription factor, transcriptome

## Abstract

Some cultivars of lettuce accumulate anthocyanins, which act as functional food ingredients. Leaf lettuce has been known to be erratic in exhibiting red color when grown under artificial light, and there is a need for cultivars that more stably exhibit red color in artificial light cultivation. In this study, we aimed to dissect the genetic architecture for red coloring in various leaf lettuce cultivars grown under artificial light. We investigated the genotype of *Red Lettuce Leaf* (*RLL*) genes in 133 leaf lettuce strains, some of which were obtained from publicly available resequencing data. By studying the allelic combination of *RLL* genes, we further analyzed the contribution of these genes to producing red coloring in leaf lettuce. From the quantification of phenolic compounds and corresponding transcriptome data, we revealed that gene expression level‐dependent regulation of *RLL1* (bHLH) and *RLL2* (MYB) is the underlying mechanism conferring high anthocyanin accumulation in red leaf lettuce under artificial light cultivation. Our data suggest that different combinations of *RLL* genotypes cause quantitative differences in anthocyanin accumulation among cultivars, and some genotype combinations are more effective at producing red coloration even under artificial lighting.

## Graphical abstract

In lettuce grown under artificial light, four *RLL* genes and *ANS* play important roles in leaf coloration. Different combinations of their genotypes produce quantitative differences in anthocyanin accumulation among cultivars.

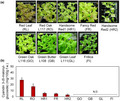

## 1 INTRODUCTION

Anthocyanins are commonly found in the plant kingdom and act as scavengers of reactive oxygen species produced by biotic and abiotic stress (Ahmed et al., 2015; Chalker‐Scott, 1999; Liu et al., 2018). In the human diet, anthocyanins are functional food components that act as antioxidants and have anti‐inflammatory effects (Pojer et al., 2013). Anthocyanin‐rich crops are widespread and popular in supermarkets, such as apples, strawberries, turnips, cabbage, and lettuce. Especially lettuce is a major crop grown in artificial light‐type plant factories. Artificial light‐type plant factories can grow vegetables regardless of abnormal weather conditions. In addition, the growing environment can be controlled for stable and high anthocyanin synthesis. This makes it possible to ensure food safety and a stable supply of functional vegetables.

Anthocyanins are secondary metabolites synthesized via the phenylpropanoid biosynthesis pathway using phenylalanine as precursor (Liu et al., 2018; Petroni & Tonelli, 2011; Saito et al., 2013). Anthocyanin biosynthesis and its regulatory mechanisms in plants have been extensively studied using molecular genetics in model plants, including *Arabidopsis*. The biosynthetic pathway involves eleven genes encoding the enzymes phenylalanine ammonia lyase (*PAL*), cinnamate 4‐hydroxylase (*C4H*), 4‐coumarate CoA ligase (*4CL*), chalcone synthase (*CHS*), chalcone isomerase (*CHI*), flavanone 3‐hydroxylase (*F3H*), flavonoid 3′‐hydroxylase (*F3*'*H*), dihydroflavonol 4‐reductase (*DFR*), anthocyanidin synthase (*ANS*), flavonoid‐3‐*O*‐glucosyltransferase (*UFGT*), and anthocyanin 6”‐*O*‐malonyltransferase (*3MaT*). Anthocyanins are then stored in the vacuole as glycosides. Glutathione *S*‐transferase (*GST*) and multidrug resistance‐associated protein (*MRP*) genes have been identified as transporters that transport anthocyanins into vacuoles (Goodman et al., 2004; Sun et al., 2012). The regulatory genes for the anthocyanin biosynthesis have also been identified. Transcriptional activation of biosynthetic genes is triggered by the MBW complex, comprising R2R3‐type MYB transcription factor, bHLH transcription factor, and WD40 factor. In most cases, the expression of MYB and bHLH regulatory genes are specific for pigmented tissues, while that of WD40 genes, which are involved in stabilizing the MBW complex, is similar in both anthocyanin‐pigmented and non‐pigmented tissues (Koes et al., 2005; Ramsay & Glover, 2005).

Four genes involved in leaf coloration of lettuce have been cloned, namely *Red Lettuce Leaf (RLL) 1* to *4* (Su et al., 2020). These four genes are genetically polymorphic, and have strong, weak, or no effects on anthocyanin accumulation, depending on the genotype (Figure S1; Type A and B, respectively). Among these genes, *RLL1*, encoding a bHLH transcription factor, and *RLL2*, encoding a MYB transcription factor, have been identified as quantitative trait loci (QTLs) responsible for leaf coloration in a red lettuce cultivar (Su et al., 2020). This implies that the regulatory network of transcription factors and other regulators for anthocyanin biosynthesis is conserved in lettuce. *RLL3* is a homolog of *MYBL2* in *Arabidopsis*. *RLL4* is a homolog of *Arabidopsis RUP1*, encoding a negative regulator of UV‐B signaling. A genome‐wide association study (GWAS) using large‐scale RNA sequencing (RNA‐seq) data sampled from the major cultivars and related species identified six candidate loci, including *RLL1*, *RLL2* and *ANS*, as expression QTLs (eQTLs) responsible for natural variations in anthocyanin content in lettuce leaves during domestication (Zhang et al., 2017).

Thus, so far, these studies have revealed that natural genetic variations are important for leaf coloration in lettuce under field conditions and discussed how the natural variations controlling leaf color were selected during domestication. However, the identity of those variations that determine anthocyanin accumulation in red lettuce cultivars remains to be elucidated.

Light is not only an essential energy source for photosynthesis but also plays a critical role as an environmental signal in plant development and physiology, such as leaf expansion, stem elongation, and metabolism (Folta, 2004; Kitazaki et al., 2018; McNellis & Deng, 1995). Anthocyanin production in plants can be increased by changing the quality and quantity of light in growth environments (Zoratti et al., 2014). In lettuce, genes whose expression level varies depending on light quality have been reported from omics analysis, and *ANS* is among them (Kitazaki et al., 2018). The *ANS* gene is the target of RLL1 and RLL2, while *RLL3* and *RLL4* are involved in the regulation of the expression of *ANS* (Su et al., 2020). However, little is known about the differences among lettuce cultivars in the genetic effects on the efficiency of anthocyanin production under artificial light. The advantage of artificial light‐type plant factories is that light quality can be controlled according to the purpose. The knowledge of these five genes is expected to be used for environmental control in plant factories. We focused on these five genes as important factors causing quantitative differences in anthocyanin accumulation.

To elucidate the genetic effects of *RLL* genes on red coloration under artificial lighting, we evaluated leaf lettuce cultivars for the core set of *RLL* genotypes. While determining the *RLL* genotype, we found a nonsense mutation in the *ANS* gene in silico by means of TASUKE (https://tasuke.dna.affrc.go.jp), a comparative genome analysis tool (Kumagai et al., 2013) that we applied to publicly available lettuce next‐generation sequencing (NGS) data and new NGS data produced in this study. From the results of *RLL* genotyping, we selected nine representative cultivars carrying different combinations of *RLL* genotypes for further quantification of phenolic compounds and transcriptome analysis. Quantitative analysis of phenolic compounds showed a genotype‐dependent accumulation of anthocyanin under a given artificial environment. Integrated analysis of anthocyanin levels and RNA‐seq data sampled from nine cultivars with different genotype combinations revealed that, in addition to anthocyanin biosynthetic genes, transcript levels of a group of genes, including *RLL1* and *RLL2*, were highly correlated with anthocyanin levels. This result indicated that the genetic network regulating MBW activity plays a critical role in anthocyanin accumulation in lettuce under artificial lighting. Our results supply a genetic basis for conferring red coloration in lettuce and not only offer a strategy for producing new lettuce cultivars containing high levels of phenolic compounds, such as anthocyanin, but also provide valuable information for lettuce‐breeding programs using genome‐sequencing data.

## 2 MATERIALS AND METHODS

### 2.1 Plant materials and growth conditions

Leaf lettuce (*Lactuca sativa* var. *crispa* L.) cv. ‘Red Leaf’, ‘Red Oak’, ‘Fancy Red’, ‘Green Oak’, ‘Green Butter’, ‘Green Leaf’, ‘Fancy Green’ (Nakahara Seed, Fukuoka, Japan), ‘Handsome Red 1’, ‘Handsome Red 2’ (Yokohama Ueki, Kanagawa, Japan), ‘Frillice’ (Snow Brand Seed, Hokkaido, Japan) were used. Seeds were sown on polyurethane foam blocks, which were imbibed in tap water and placed into stainless steel trays. They were maintained at 23°C / 18°C (day/night) and 70–85% relative humidity, under 180 μmol m^−2^ s^−1^ white fluorescent light and long day conditions (16 h light / 8 h dark) in a growth chamber. One‐week‐old seedlings in urethane foam blocks were inserted into plug trays (3 cm × 3 cm × 4 cm) and grown hydroponically with 0.5 unit of Otsuka A formula (electrical conductivity 1.35–1.6 dS m^−1^, pH 5.5–6.5, OAT Agrio, Tokyo, Japan).

### 2.2 *RLL* genotyping

To genotype the *RLL* locus, a polymerase chain reaction (PCR)‐based assay was performed using genomic DNA as a template with the KAPA2G Fast ReadyMix PCR kit (Kapa Biosystems) in accordance with the manufacturer's instructions. For *RLL1*, we used a primer that identifies a 5‐bp deletion; for *RLL3* and *RLL4*, we used dCAPs primers with *Mbo*I and *Hinc*II restriction enzyme sites, respectively, that identify a 1‐bp substitution (Su et al., 2020). For *RLL2*, we designed a primer to identify the 15‐bp deletion, and for *ANS*, we used a dCAPs primer designed to create a *Mbo*I site at the 1‐bp substitution GAG→TAG (stop), which we found by aligning the genome‐sequencing data of 88 cultivars. The PCR products and their digests with restriction enzymes were separated by agarose gel electrophoresis and fragment sizes were checked except for *RLL1*. The genotype of *RLL1* was determined by whether or not the target sequence was amplified by PCR.

### 2.3 Genome sequencing using NGS and analysis with TASUKE

Young leaf tissues were powdered with liquid N_2_ and total genomic DNA was extracted with the DNeasy Plant Mini kit (Qiagen) in accordance with the manufacturer's instructions. The quality of DNA samples was verified by BioAnalyzer. The DNA libraries were sequenced using the Illumina HiSeq 2000, Genome Analyzer IIx or HiSeq X instruments (Illumina Co., Ltd.), and paired‐end reads were obtained. All reads were mapped against *L. sativa* cv. ‘Salinas’ genome (Reyes‐Chin‐Wo et al., 2017) pseudomolecules using BWA‐MEM (Li & Durbin, 2009), and duplicates were removed using picard MarkDuplicates (http://broadinstitute.github.io/picard/). Variants were called using GATK GenotypeGVCFs (Van der Auwera and O'Connor 2020) and filtered using GATK VariantFiltration with settings QD <2.0 || FS > 60.0 || MQ < 40.0 || MQRankSum < −12.5 || ReadPosRankSum < −8.0. The variants for each lettuce cultivar were displayed using the TASUKE genome browser (Kumagai et al., 2013).

### 2.4 Analysis of phenolic compounds

The relative content of phenolic compounds in each extract was determined by a method described by Arapitsas et al. (2008) with minor modifications. Powdered lettuce samples (0.05–0.1 g) were extracted with 250–500 μl (proportional to the frozen weight) of methanol–water–formic acid solution (40:59:1, v/v/v) for 1 h after 3‐min sonication, followed by two extractions with 250 μl 80% methanol. The combined extract was centrifuged at 13,000 *g* for 5 min. The supernatant was filtered through a 0.45‐μm centrifugal filter (Merck‐Millipore) and the filtrate made up to 1 ml. Ten μl of the resultant extract was injected into the HPLC system that comprised an Agilent Technologies 1100 Series HPLC (Agilent) equipped with a YMC‐Triart C18 column (150 mm × 2.0 mm I.D., S‐3 μm, 12 nm; YMC Co. Ltd.). The flavonoids and anthocyanins in the extract were separated with a mobile phase (0.2 ml min^−1^) consisting of (A) 5% (v/v) formic acid aqueous solution and (B) acetonitrile, according to a multistep solvent gradient program (Table S1) at 28°C. The anthocyanins in the eluent were monitored at 520 nm, while the other flavonoids and phenolic compounds were detected at 350 nm. The identification of phenolic compounds from the measured HPLC peaks was conducted according to their UV spectra, retention times and electrospray ionization tandem mass spectrometry (ESI–MS/MS, Ultimate3000 RSLC system, Thermo Fisher Scientific). Phenolic compounds were quantified using calibration curves of the following chemicals: chicolic acid and chlorogenic acid (Toronto Research Chemicals, Toronto, Canada); quercetin‐3‐*O*‐glucoside (Extrasynthese, Genay, France); cyanidin 3‐*O*‐glucoside (Nagara Science). Abundance of cyanidin derivatives and quercetin derivatives was calculated as an equivalent amount of quercetin‐3‐*O*‐glucoside and cyanidin 3‐*O*‐glucoside, respectively.

### 2.5 Quantitative real‐time PCR

Frozen samples were powdered with liquid N_2_ and RNA was extracted with the RNeasy Plant Mini kit (Qiagen) in accordance with the manufacturer's instructions. RNA (500 ng) was subjected to cDNA synthesis using the ReverTra Ace cDNA synthesis kit with genome remover (TOYOBO). qRT–PCR was performed with the qPCR MasterMix SYBR reagent (TOYOBO) using primers listed in Table S2 and detected by ViiA7 (Thermo Fisher, Applied Biosystems). The cycle program for the PCR was according to the manufacturer's instructions.

### 2.6 Plasmid construction and agroinfiltration

Full‐length cDNAs of *RLL1*, *RLL2* and *LsTTG1* were amplified by *PrimeSTAR* GXL DNA polymerase according to the manufacturer's instructions (TAKARA). Amplified DNA fragments were verified by sequencing. Each full‐length cDNA was then amplified by individual primers with the linker sequence (Table S2) and cloned into the binary vector, pMLH7133 (Mochizuki et al., 2003), digested with *Bam*HI and *Sac*I by means of the infusion‐HD system (TAKARA). Expression of each gene was regulated by the 7‐fold replicated enhancer of the cauliflower mosaic virus 35S promoter (E7) with the tobacco mosaic virus omega sequence insertion and the first intron of a gene for phaseolin.

The plasmids harboring each cDNA were transformed into *Agrobacterium* strain GV3101 by electroporation. Resulting recombinant *Agrobacteria* were separately cultured overnight in liquid media containing 50 mg l^−1^ kanamycin. Cultured cells were resuspended in agroinfiltration buffer (10 mM MES–KOH, pH 5.7, 10 mM MgCl_2_) and adjusted to high and low concentration based on absorbance (OD_595_ = 0.9 and 0.4, respectively). For suppressing the interfering activity of endogenous RNA, *p19* (Lakatos et al., 2004) was co‐infiltrated at OD_595_ = 0.1. After incubation for 3 h at room temperature, *Agrobacterium* solutions were infiltrated into leaves of cv. ‘Fancy Green’ by needleless 1‐ml syringe. Leaf samples were collected at 24 h after infiltration.

### 2.7 RNA sequencing

Total RNA was extracted from frozen and ground leaves using the RNeasy Plant Mini kit (Qiagen) according to the manufacturer's instructions. RNA‐seq libraries were prepared using the NEBNext Ultra II Directional mRNA‐seq kit (New England Biolabs) according to the manufacturer's instructions. RNA‐seq libraries were sequenced using the Illumina NovaSeq 6000 at Macrogen Japan. Sequencing of libraries was performed on the S4 flow cells with paired‐end 150‐bp and unique dual index reads.

### 2.8 Data processing

Raw reads were filtered to remove adapters and low quality reads with Trimmomatic v0.39 (Bolger et al., 2014) and the quality of the data was checked with FastQC_v0.11.9 (Andrews, 2010). The clean reads were mapped to the *L. sativa* cv. ‘Salinas’ genome (Reyes‐Chin‐Wo et al., 2017) using HISAT2 v2.2.1 (Kim et al., 2015). Gene expression level was calculated based on the transcripts per million (TPM) method in StringTie v2.1.5 (Pertea et al., 2015).

## 3 RESULTS

### 3.1 Categorization of leaf lettuce cultivars bred in field conditions according to *RLL* genotype

**FIGURE 1:**
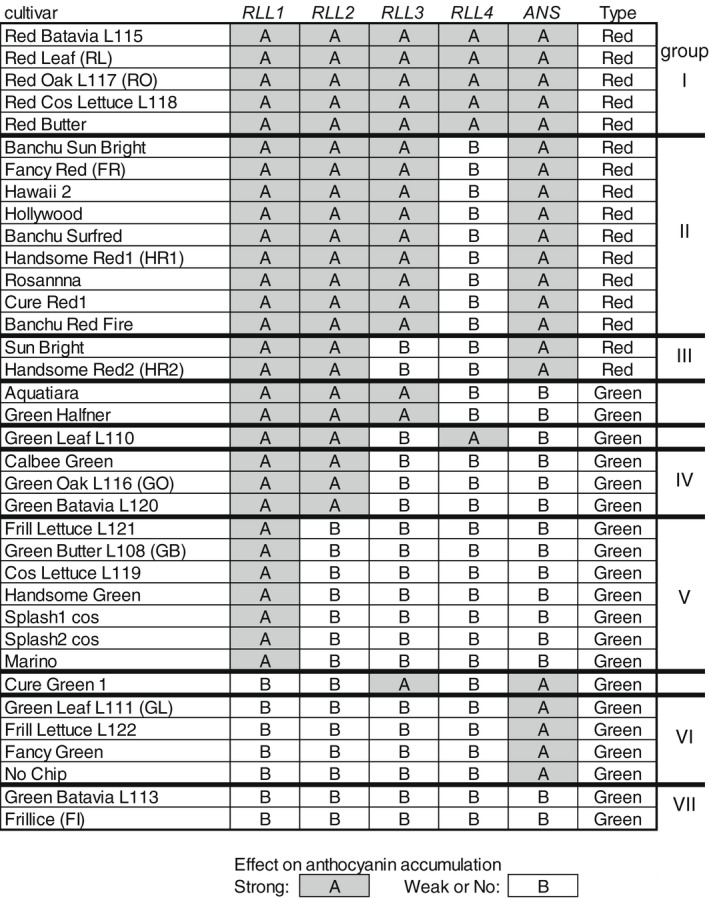
*RLL* genotype in 36 leaf lettuce cultivars sold in Japan. Genotyping was performed using PCR with primers designed for discriminating the functional nucleotide substitution in individual genes. Gray and white indicate genotypes with strong (A) and weak or no (B) effects on anthocyanin accumulation, respectively. Detailed information about the nucleotide polymorphisms is shown in Figure S1. The genotypes were grouped from I to VII, according to the combination of genotypes, and one or two cultivars from each group were selected for subsequent experiments.

Su et al. (2020) cloned four polymorphic genes controlling leaf coloration in lettuce by QTL‐seq using a biparental F2 population (*RLL1* to *RLL4*; Figure S1), and Zhang et al. (2017) analyzed RNA‐seq data and identified *ANS* as an eQTL associated with flavonoid biosynthesis. After identifying the functional nucleotide polymorphism (FNP) in *ANS*, as shown below, we first investigated all *RLL* genotypes in 36 leaf lettuce cultivars sold in Japan by means of primers designed for discriminating the FNPs in individual genes. In *RLL2*, there is a tandem duplication of the R2R3‐type *MYB* gene in a red leaf lettuce cultivar (*RLL2A*, *RLL2B*; Figure S1b, Type A) (Su et al., 2020). Only one gene (*RLL2A*) was actively expressed in red leaf cultivars (Figure S2). To evaluate *RLL2* genotypes, we used a structure variant (15‐bp in/del) in the last exon of both red and green cultivars of the *RLL2* genes (Figure S1). As shown in Figure 1, the red leaf lettuce cultivars contained the functional type (type A) of *RLL1*, *RLL2*, and *ANS* alleles, indicating that these alleles are important for leaf lettuce to turn red under field conditions. Additional variations producing a specific amino acid substitution in *RLL3* and *RLL4* genes in red leaf lettuce cultivars were likely to influence quantitative differences in their red coloration among these cultivars, because functional versions of these genes are not necessary for red coloration in leaves. In contrast, green leaf lettuce cultivars harboring functional forms of only *ANS* or *RLL1* and *RLL2* were frequently observed. Our results indicate that the FNPs of *RLL* genes identified in the previous studies (Su et al., 2020) can generally be used for the selection of leaf coloration and suggest that combinations of these alleles might unintentionally have occurred during breeding performed in natural field conditions.

### 3.2 Confirmation of the *RLL* genotype of lettuce from around the world using NGS data

**FIGURE 2:**
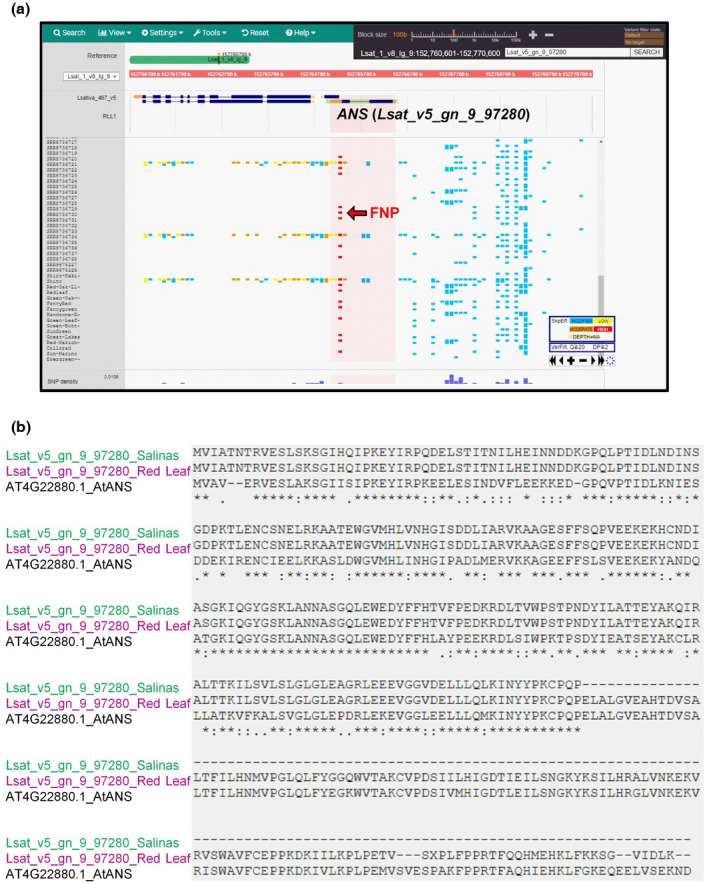
Visualization of *ANS* genotype in lettuce accessions. (a) Visualization of Lsat_v5_gn_9_97280 as *ANS* in the TASUKE system. The likely effects of sequence variations detected in next‐generation sequencing data can be displayed in this system. The effects on annotated genes are estimated by SnpEff, which classes their impact as high, moderate, low and modifier (Cingolani et al., 2012), represented in TASUKE as red, orange, yellow, and blue, respectively. High impact refers to (e.g.) truncations, large in/dels or introducing/losing stop codons. Moderate impact refers to other non‐synonymous coding region changes. Low impact refers to synonymous changes. Modifier refers to changes in non‐coding genic regions. (b) Alignment of three ANS polypeptide sequences from lettuce and *Arabidopsis*. Truncated and intact versions of ANS proteins in lettuce are indicated by green and red letters, respectively.

To check the distribution of the known *RLL* genotype in other lettuce accessions, we used genome data collected from diverse lettuce accessions obtained around the world. The sequence variations detected in NGS data were visualized by a comparative genome analysis tool, TASUKE, that enables the user to effectively visualize variations in nucleotide substitutions and in/dels in collections of individually annotated genes (Kumagai et al., 2013). In addition to using NGS data for 88 accessions that are publicly available, we implemented TASUKE with newly added NGS data for nine cultivars used in this study and nine accessions available from the NARO Genebank (https://www.gene.affrc.go.jp/index_en.php). Although the reference *L. sativa* cv. ‘Salinas’ genome had a nonsense mutation in the *ANS* gene (Lsat_v5_gn_9_97280; Figure 2a), lettuce accessions with red coloration had no mutation in the exon, producing an intact polypeptide of ANS with high similarity to the *Arabidopsis* ANS protein (Figure 2b). In silico genotyping for four *RLL* genes further supported the notion that three functional genotypes, for *RLL1*, *RLL2*, and *ANS*, constituted a core set for red coloration (Figure S3). However, by expanding the investigation of the *RLL* genotype into lettuce accessions from around the world, we found an exception to the association between the *RLL* genotype and leaf coloration in the cultivar, ‘Flashy Trout Back’. Because ‘Flashy Trout Back’ has been described as having green leaves with red speckles, anthocyanin biosynthesis is potentially active and might be regulated spatially in this cultivar. In addition, we found a putative FNP of *GST* (Lsat_1_v5_gn_3_87760) that has been identified as a candidate for one e‐QTL (Zhang et al., 2017, Figure S1). The highly conserved amino acid, Pro^43^, in the N‐terminal region was converted into Ser^43^. The N‐terminal region of GST is required for glutathione binding (Dixon et al., 2002).

### 3.3 Profiles of phenolic compounds in leaf lettuce cultivars with different combination of the *RLL* genotype grown under artificial light

**FIGURE 3:**
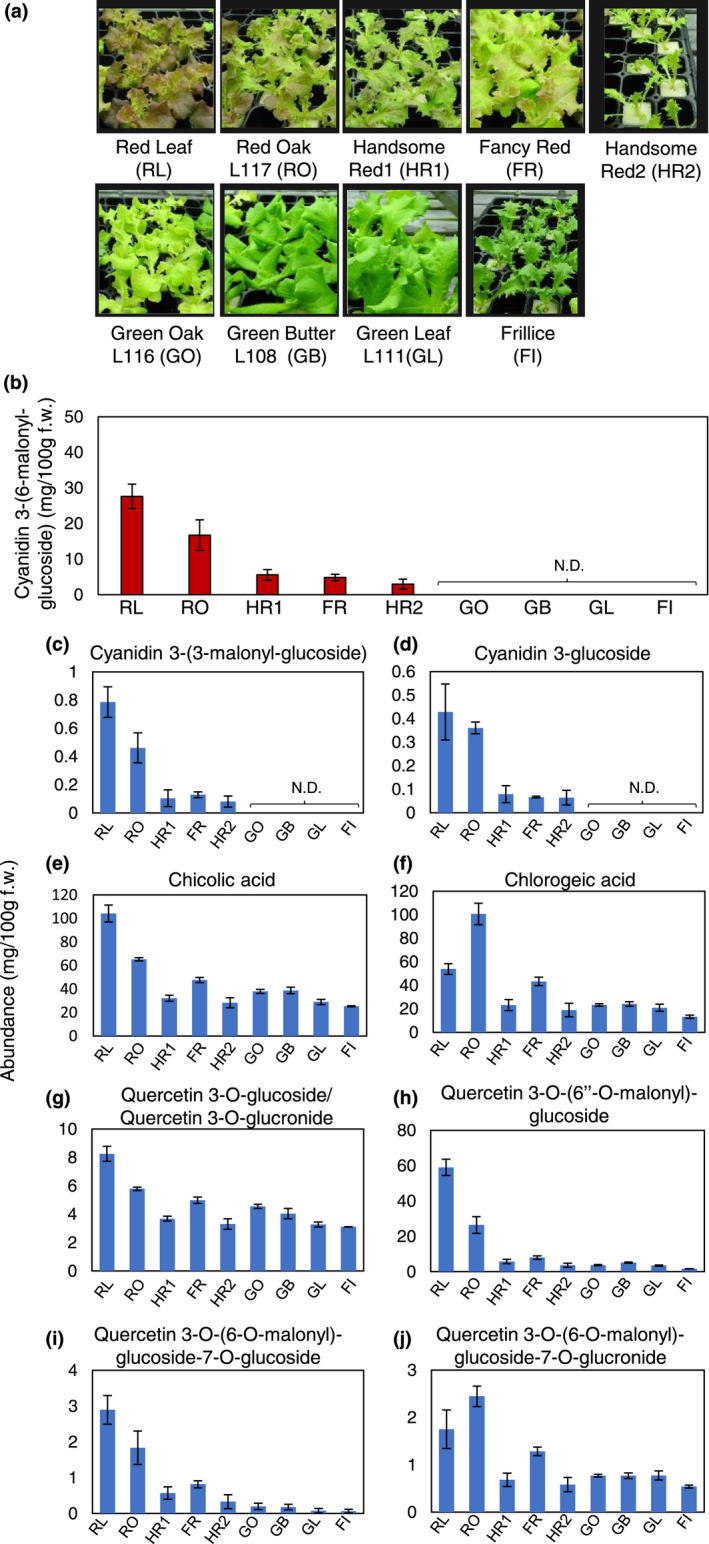
Abundance of phenolic compounds, including anthocyanin. (a) Gross morphologies of 17‐day‐old plants grown under fluorescent light. (b–j) Abundance in nine leaf lettuce cultivars of cyanidin‐3‐*O*‐(6′′‐*O*‐malonyl)‐glucoside (b), cyanidin 3‐*O*‐(3′′‐*O*‐malonyl)‐glucoside (c), cyanidin 3‐*O*‐glucoside (d), chicolic acid (e), chlorogenic acid (f), quercetin 3‐*O*‐glucoside/quercetin 3‐*O*‐glucuronide (g), quercetin 3‐*O*‐(6′′‐*O*‐malonyl)‐glucoside (h), quercetin 3‐*O*‐(6′′‐*O*‐malonyl)‐glucoside‐7‐*O*‐glucoside (i) and quercetin 3‐*O*‐(6′′‐*O*‐malonyl)‐glucoside‐7‐*O*‐glucronide (j). Cultivars are indicated as RL (Red Leaf), RO (Red Oak L117), HR1 (Handsome Red 1), FR (Fancy Red), HR2 (Handsome Red 2), GO (Green Oak L116), GB (Green Butter L108), GL (Green Leaf L111), FI (Furillice). Data are means ± SE (*n* = 3). N.D. denotes ‘not detected’.

Green leaf lettuce has been grown commercially in controlled environments to maximize yield in artificial light‐type plant factories. Additional light treatment, such as blue or UV light, can enhance the red color of red leaf lettuce (Ebisawa et al., 2009; Goto et al., 2016; Shoji et al., 2010), implying that stable production of red leaf lettuce with high anthocyanin contents might be difficult in closed‐type plant factories. Our genotyping results suggested that differences in the *RLL* functionality might cause quantitative variation in anthocyanin accumulation among leaf lettuce cultivars. Therefore, we quantified phenolic compounds, including anthocyanin, in different leaf lettuce cultivars grown under fluorescent light. We selected five red leaf lettuce cultivars and four green leaf lettuce cultivars according to the combination of *RLL* genotype (group I to VII, shown in Figure 1). In our growth conditions, the degree of red coloring in leaves of the selected five red leaf cultivars seemed to be consistent with the combination of the genotype with a strong effect on increasing anthocyanin content in natural light (Figure 3a). To confirm this quantitatively, anthocyanin and related phenolic compounds were fractionated by high performance liquid chromatography (HPLC) and then measured by absorbance with diode array detection (DAD). In our analysis, cyanidin‐3‐*O*‐(6′′‐*O*‐malonyl)‐glucoside was detected as major anthocyanin in leaf lettuce. In red leaf cultivars, cyanidin‐3‐*O*‐(6′′‐*O*‐malonyl)‐glucoside and its analogs accumulated in a *RLL* genotype‐dependent manner (Figure 3b–d). Presence of the reduced function‐type *RLL4* allele significantly enhanced anthocyanin accumulation in our growth conditions. Interestingly, anthocyanin accumulation was consistently higher in ‘Red Leaf’ (RL) than in ‘Red Oak L117’ (RO), although both cultivars exhibit the same genotype of known *RLL* genes (Figure 1). In addition to anthocyanin, we analyzed other phenolic compounds, that is *di*‐*O*‐caffeoyltartaric acid (chicoric acid), 3‐trans‐*O*‐caffeoylquinic acid (chlorogenic acid), quercetin glucosides, quercetin glucuronides, caffeoylmalic acid, and caffeoyltartaric acid (Figure 3e–j, Figure S4). Other flavonoids, namely naringenin, kaempferol, and leucocyanidin and their glycosides, were not detected, possibly because the content was below detectable levels. A similar tendency to that found for anthocyanin accumulation in the five red cultivars and four green cultivars was generally observed for contents of chicoric acid, quercetin‐3‐*O*‐(6′′‐*O*‐malonyl)‐glucoside, quercetin‐3‐*O*‐(6′′‐*O*‐malonyl)‐glucoside‐7‐*O*‐glucroside, and chlorogenic acid. However, chicoric acid and chlorogenic acid contents in the green leaf cultivars, ‘Green Oak L116’ (GO) and ‘Green Butter L108’ (GB), were higher than in the red leaf cultivars, ‘Handsome Red 1’ (HR1) and ‘Handsome Red 2’ (HR2). Derivatives of caffeic acid seemed to be accumulated regardless of combination of the *RLL* genotype (Figure S4). Even though the green leaf cultivar, ‘Green Leaf L111’ (GL), possesses the functional *ANS* gene, we found no detectable anthocyanin (Figure 3b–d). In contrast, although GO possessed both functional *RLL1* and *RLL2*, anthocyanin was not detected consistently because anthocyanin biosynthesis is stopped by *ANS* deficiency in the penultimate step of the pathway. Thus, we conclude that the core set of functional *RLL1*, *RLL2*, and *ANS* genes confer the capability for anthocyanin biosynthesis in red leaf lettuce cultivars; in addition, the *RLL3* and *RLL4* genotype is able to enhance activity of the MBW complex for anthocyanin biosynthesis. Interestingly, presence of functional *RLL1* and *RLL2* in GO was insufficient to produce anthocyanins or flavonols in green leaf lettuce cultivars. It should be noted that final step in anthocyanin biosynthesis also seem to be required for accumulating flavonols in leaf lettuce.

### 3.4 Expression of *RLL* genes in leaf lettuce cultivars

**FIGURE 4:**
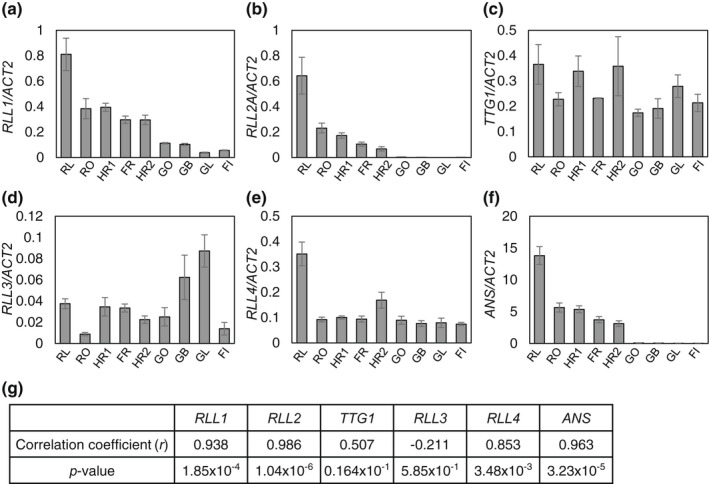
Expression of four *RLL*, *ANS* and *LsTTG1* genes in five red leaf lettuce cultivars (RL, RO, HR1, FR, HR2) and four green leaf lettuce cultivars (GO, GB, GL, FI). Transcript levels were quantified by qRT–PCR using *LsActin2* (*ACT2*) as an internal control. Relative transcript levels of *RLL1* (a), *RLL2A* (b), *TTG1* (c), *RLL3* (d), *RLL4* (e) and *ANS* (f) are shown. Data are means ± SD (*n* = 3 or 5). Pearson's correlation coefficient was calculated between anthocyanin levels and transcript levels for each gene (g).

The results of quantification of phenolic compounds in the nine cultivars indicated that, in a given environmental condition, cumulative gene effects conferred by appropriate *RLL* genotypes would increase anthocyanin contents. To investigate the fundamental mechanism of anthocyanin accumulation, we analyzed the expression of individual *RLL* genes by qRT–PCR (Figure 4). We also analyzed the lettuce *TTG1* (*LsTTG1*) gene because it is involved in the MBW complex. Levels of *RLL1* and *RLL2* expression were upregulated in red leaf cultivars (Figure 4a,b), corresponding to a quantitative effect on anthocyanin accumulation (Figure 3b). The induction of gene expression of *RLL1* and *RLL2* was remarkably high in cultivar RL. In contrast, the expression of *LsTTG1*, a homolog to *Arabidopsis TTG1*, was not affected by the status of anthocyanin accumulation in the nine cultivars (Figure 4c). The expression of *RLL3*, which encodes a suppressive MYB factor for anthocyanin accumulation, showed weak inverse correlation with anthocyanin levels and was low in cultivar RO (Figure 4d). The expression of *RLL4* was relatively low in eight cultivars but higher in RL under our artificial growth conditions (Figure 4e). Consistent with the previous study on *Arabidopsis* (Gonzalez et al., 2008), the expression of *ANS* was highly correlated with that of *RLL1* and *RLL2*, the components of the MBW complex acting as the direct transcriptional activator (Figure 4f). The expression levels of functional *RLL1* and *RLL2* genes were strongly correlated with and are likely to be major determinants of the content of anthocyanin under artificial environments, resulting in variation of anthocyanin contents among red leaf lettuce cultivars (Figure 4g).

**FIGURE 5:**
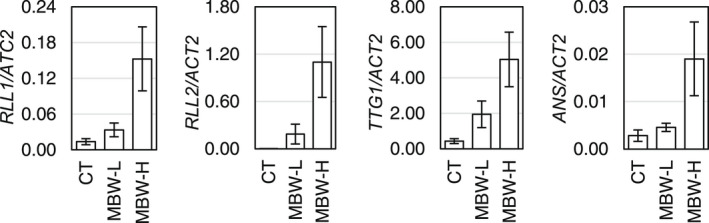
Transcript levels of genes induced by transient assay. Low (MBW‐L) and high (MBW‐H) concentrations of *Agrobacterium* carrying the *35S::RLL1*, *35S::RLL2* and *35S::LsTTG1* constructs or *p19* only (control, CT) were mixed and then infiltrated into leaves of lettuce cv. ‘Fancy Green’. Transcript levels of *RLL1*, *RLL2*, *TTG1*, and *ANS* were quantified by qRT–PCR using *LsActin2* (*ACT2*) as an internal control. Data are means ± SD (*n* = 3).

To test our hypothesis that the expression levels of *RLL1* and *RLL2* correlate with that of *ANS*, we set up transient expression in leaves of ‘Fancy Green’ leaf lettuce using agroinfiltration. Transfection with *RLL1*, *RLL2*, and *LsTTG1* at two different concentrations successively produced a dosage‐dependent expression of *RLL1* and *RLL2* in lettuce leaves of the same genetic background and demonstrated the MBW dosage‐dependent expression of the downstream *ANS* gene (Figure 5).

### 3.5 Expression profiles of genes associated with anthocyanin accumulation in leaves

**FIGURE 6:**
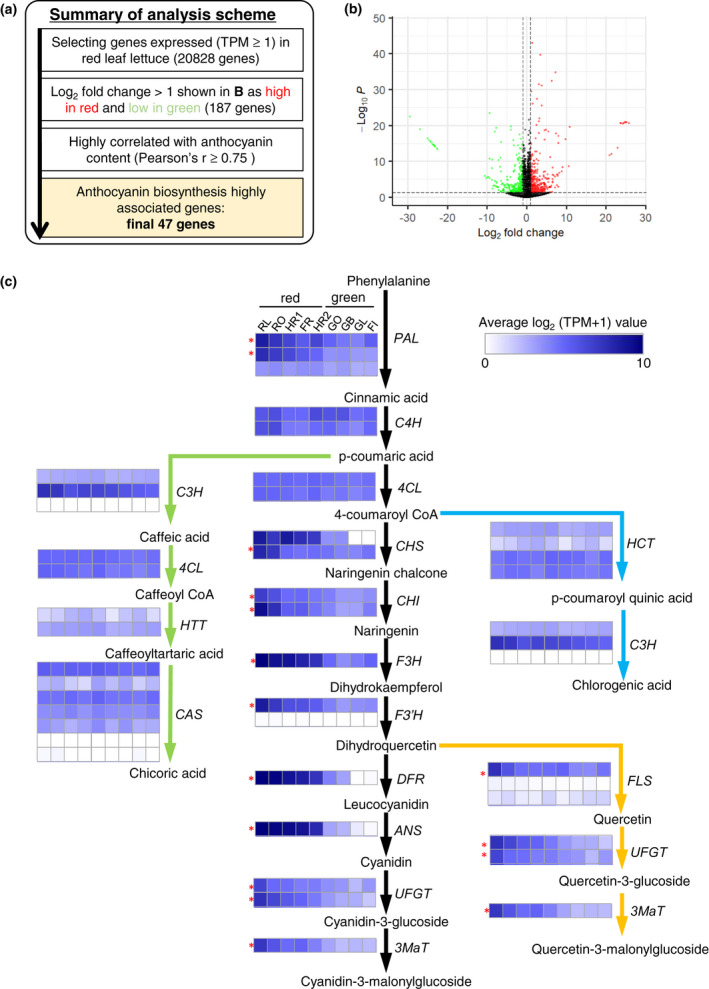
Analysis of RNA‐seq data. (a) Summary of analysis scheme for RNA‐seq data. (b) Volcano plot of RNA‐seq data showing the differentially expressed genes (DEGs) between red and green leaf lettuce cultivars. Red and green dots indicate up‐regulated and down‐regulated DEGs, respectively (*p* < 0.05, log_2_ fold change >1). (c) Mapping of expression profiles onto the anthocyanin biosynthesis pathway in leaf lettuce. Each colored box shows the average log_2_ (TPM + 1) values of a biosynthetic pathway gene, according to the color scale. Genes selected as anthocyanin biosynthesis highly associated genes are indicated with asterisks (also indicated in Figure S5). Branched pathways for biosynthesis of chlorogenic acid (blue arrows), chicoric acid (green arrows), and quercetin glucosides (orange arrows) are indicated.

As described above, our results from qRT–PCR showed that transcriptional regulation of both *RLL1* and *RLL2* was critical in controlling the final anthocyanin content of lettuce leaves. To find genes related to anthocyanin accumulation, we performed RNA‐seq analysis using the same samples as those used for quantification of phenolic compounds (Figure 3). After normalizing the data, 38,910 genes were assembled. Of these, 20,828 genes showed expression (TPM ≥1) in all the red leaf lettuce cultivars. Among those genes, we chose genes whose expression was significantly more than twice as high in five red leaf lettuce cultivars as in four green leaf lettuce cultivars: 187 genes were extracted by the Wald test of DESeq2 as statistically significant (*p* < 0.05, Figure 6a,b). Because *RLL1* and *RLL2* expression was highly correlated with anthocyanin levels (Figure 4), we performed co‐expression analysis between the expression of those 187 genes and cyanidin‐3‐*O*‐(6′′‐*O*‐malonyl)‐glucoside levels using all biological replicate samples and identified 47 genes with a high Pearson's correlation coefficient (*r* ≥ 0.75) as anthocyanin biosynthesis highly associated genes (ABHAG; Figure S5). The selected 47 genes were then allocated into five clusters using the *k*‐means method. As we expected, the 47 genes included *RLL1* and *ANS*. Genes in clusters 1, 2, and 3 had a broad range of phenotype from high to low anthocyanin content and the detected absolute values of gene expression were higher than those of clusters 4 and 5. Clusters 1 to 3 contained two *PAL* genes (the first step of the phenylpropanoid biosynthetic pathway), the entire series of anthocyanin biosynthetic genes, *FLS* (flavonol biosynthetic gene), and two *GST* genes (anthocyanin transporters), indicating that genes in clusters 1–3 play central roles in the biosynthesis and accumulation of phenolic compounds. In contrast, clusters 4 and 5 had relatively low expression levels and contained several known transcription factors, namely *AtMYB12* homolog, *AtMYB75/AtPAP* homolog, and *AtNAC098/CUC2* homolog, whose activities are associated with biosynthesis of phenolic compounds (Borevitz et al., 2000; Ma et al., 2021; Mahmood et al., 2016; Wang et al., 2016). These genes were thought to act as enhancers for biosynthesis and/or accumulation of phenolic compounds in lettuce. Clusters 4 and 5 also contained some abiotic stress response genes, such as peroxidase, UVR3, and LRR family protein (homolog of cold‐regulated gene AT3G19320; Vergnolle et al., 2005). In addition, genes encoding *TT8*, Ser/Thr kinase, and cryptochrome, which have been previously identified as genes co‐expressed with flavonoids in *Arabidopsis* (Yonekura‐Sakakibara et al., 2008), were among the genes in clusters 4 and 5.

We mapped the expression profiles of phenylpropanoid biosynthetic genes onto the biosynthetic pathway (Figure 6c). Our results confirmed that the expression profiles of the series of anthocyanin biosynthetic genes were well coordinated with the accumulation of anthocyanin across different cultivars. Step of flavonol biosynthesis was also co‐regulated with anthocyanin biosynthesis. In contrast, genes involved in the chlorogenic acid and chicoric acid biosynthetic pathways appeared to be expressed independently of anthocyanin biosynthesis. Our results indicate that the ABHAG might be used as biomarkers to precisely monitor a status of production of not only anthocyanin but also other phenolic compounds in a certain cultivar or in a particular artificial environment.

## 4 DISCUSSION

With the increasing threat of unpredictable or extreme weather conditions, closed‐type plant factories with artificial light are commercially attractive as they can stably cultivate high quality vegetables with desirable nutritional value and functional components. Our evaluation of levels of phenolic compounds in leaf lettuce cultivars grown in a controlled environment with fluorescent light showed that accumulation of anthocyanins was dependent on the combination of known *RLL* genotypes that control red coloration. In addition, transcriptome analysis indicated that the accumulation of anthocyanin coincided with changing transcriptional patterns related to anthocyanin biosynthesis and transport, and abiotic stress responses. By combining the known genetic framework for anthocyanin biosynthesis in plants with targeted metabolomic and transcriptome data, we revealed that a dosage‐dependent regulation of *RLL1* (*bHLH*) and *RLL2* (*MYB*), which encode core components of the MBW complex, is the underlying mechanism for enhancing accumulation of anthocyanins in leaf lettuce grown under artificial light.

### 4.1 Potential relationship between anthocyanin accumulation and light intensity or quality

The red color observed in artificial light conditions is much lighter than the red color seen in the open field. The light red color is likely caused by artificial light in growth chamber. The reason for this is probably that light quality and light intensity are different between open field and artificial light condition. As for light intensity, homolog genes encoding ELIP1 (AT3G22840) and ubiquinone methyltransferase (AT2G41040) in ABHAG were reported to respond to high‐intensity light in Arabidopsis (Kleine et al., 2007). They showed that the induction of *ELIP1/2* expression is mediated via *CRY1* in a blue light intensity‐dependent manner. In addition, the ubiquinone methyltransferase misregulated in response to HL in the *cry1* mutant. Under our experimental conditions, lettuce *CRY1* homologs (Lsat_1_v5_gn_1_3881 and Lsat_1_v5_gn_9_41700) were expressed but not included in ABHAG because the Log_2_ fold change of the red variety compared to the green variety was less than 1.

Regarding light quality, exposure to additional blue light and UV light can enhance accumulation of anthocyanin in a red leaf cultivar (Ebisawa et al., 2009; Goto et al., 2016; Ma et al., 2021; Shoji et al., 2010). Our study revealed that the potential for anthocyanin biosynthesis in individual cultivars might be determined by the presence of particular genotypes. Comparison between genotype and contents of phenolic compounds indicated that a functional difference in the *RLL4* gene was important under our experimental conditions (Figures 1 and 3). Presence of the reduced function‐type *RLL4* allele was associated with remarkable accumulation of phenolic compounds in the cultivars RL and RO. Because *RLL4* encodes an *Arabidopsis* WD40 protein, *RUP1*, which is a negative regulator for UV‐B signaling through inhibition of the function of a UV photoreceptor, UVR8 (Gruber et al., 2010; Heijde & Ulm, 2013), a point mutation in the conserved WD40 domain of RLL4 causing the reduced function might lead to high UV sensitivity in leaf lettuce cultivars. In fact, the fluorescent light that we used included the UV‐B range within its spectrum. Therefore, it is predicted that the remarkable accumulation of phenolic compounds observed in RL and RO was caused by a higher sensitivity to UV irradiation in fluorescent light in those cultivars than that in other cultivars used in this study. Light manipulation in artificial environments using various leaf lettuce cultivars and the collection of quantitative data for content of phenolic compounds would be a worthwhile avenue for further research. Exploiting differences in the UV‐sensitivity of lettuce cultivars might offer opportunities to enhance anthocyanin accumulation under artificial light, where UV is weaker than in the field.

### 4.2 Enhancement of accumulation of anthocyanin and flavonoid compounds in leaf lettuce

Our results confirmed that the evolutionally conserved MBW complex acts as a central regulator for anthocyanin accumulation in leaf lettuce (Figure 1) and indicated that the regulation of the MBW complex activity is a major determinant for transcriptional levels of ABHAG, including anthocyanin biosynthesis and transporter genes, hence determining final anthocyanin levels (Figures 3, 4, 5). Our results showed that the combination of *RLL3* and *RLL4* alleles affects the accumulation of anthocyanin. Functionality of *RLL4* might indirectly affect the transcription levels of *RLL1* and *RLL2*. As described above, *RLL4* encodes a *RUP1* homolog in *Arabidopsis* (Gruber et al., 2010). *RUP1* negatively regulates the UV‐dependent expression of *HY5*, responsible for anthocyanin biosynthesis through transcriptional activation of the downstream transcription factor, *MYB75/AtPAP1*, a homolog of *RLL2* in lettuce (Shin et al., 2013). Thus, we consider that the reduced function of the weak *RLL4* allele cannot negatively regulate the transcriptional activation of *RLL1* and *RLL2* in response to fluorescent light, resulting in high accumulation of anthocyanin under this condition. In contrast, functional *RLL3* is able to influence anthocyanin levels through competition with the intact MBW complex formation by ejecting RLL2 from the MBW complex (Su et al., 2020).

### 4.3 Additional transcriptional regulators associated with anthocyanin biosynthesis

Among ABHAG selected in this study, three transcriptional factors in addition to *RLL1* and *RLL2* were identified (Figure S5). The first, Lsat_v5_gn_2_124061, encoded a homolog of *MYB75/AtPAP1* in *Arabidopsis* that can activate genes in both early and late steps of anthocyanin biosynthesis through the formation of the MBW complex (Gonzalez et al., 2008; Shi & Xie, 2010). The second, Lsat_1_v5_gn_9_181, encoded a *MYB12* homolog in *Arabidopsis* acting independently of the MBW complex. The third, Lsat_1_v5_gn_7_104460, is an *Arabidopsis NAC098/CUC2* homolog. Although involvement of *CUC2* itself has not been reported, the *BLOOD* (*BL*) gene, which has the highest identity to *CUC3* and is functionally redundant with its homologs *CUC1* and *CUC2*, controls activation of anthocyanin biosynthesis in blood‐fleshed peach (Zhou et al., 2015). Several studies have implicated *NAC* genes in the regulation of anthocyanin biosynthesis (Ma et al., 2021). Therefore, it is possible that MBW‐dependent expression of these transcription factors is required for stable production of phenolic compounds in certain genetic backgrounds of red leaf lettuce.

### 4.4 Phenolic compounds and their biosynthesis in leaf lettuce

The profiles of phenolic compounds in green and red oak‐leaf lettuce have been extensively characterized by ultra‐high‐performance liquid chromatography coupled online to DAD, electrospray ionization, and QToF–MS systems (Viacava et al., 2017). Consistent with those profiles, cyanidin 3‐(6‐malonylglucoside), cyanidin 3‐(3‐malonylglucoside), and cyanidin 3‐glucoside were detected only in red leaf lettuce cultivars (Figure 3b–d). Apigenin‐glucoside, identified only in the green oak‐leaf cultivar (Viacava et al., 2017), was not detected as a major peak in any green leaf cultivars used in this study.

Accumulation of high anthocyanin content can coordinately increase phenolic compounds, including flavonoids. Our quantitative analysis revealed that chlorogenic acid and chicoric acid were major phenolic compounds in leaf lettuce cultivars (Figure 3). Both phenolic compounds have long been of interest for human health because of their antioxidant effects and role in suppressing fat accumulation (Lee & Scagel, 2013; Tungmunnithum et al., 2018). Among nine cultivars with different *RLL* genotype combinations, the cultivars RL and RO, both harboring genotype combinations conducive to anthocyanin accumulation, contained not only higher anthocyanin but also higher phenolic compounds than other genotype combinations. Consistent with our data for lettuce, in potato, chlorogenic acid was the most abundant phenolic compound and was present in high levels in high‐anthocyanin cultivars (Navarre et al., 2011; Valiñas et al., 2017). Our transcriptome analysis further suggested that full activation of the entire anthocyanin biosynthesis pathway increases chlorogenic acid and chicoric acid contents as by‐products (Figure 6c). Therefore, basically, two compounds are constantly synthesized regardless of the *RLL* genotype (Figure 3e–f). But, in cultivars in which anthocyanin biosynthesis is highly active, such as RL and RO in this study, de novo *p*‐coumaric acid and 4‐coumaronyl‐CoA production would be expected to be increased, so that the precursor for chlorogenic acid and chicoric acid biosynthesis is more highly available.

Our analyses identified quercetin glucosides as the main type of flavonoid in leaf lettuce. The levels of the major quercetin glucoside (quercetin‐3‐*O*‐(6′′‐*O*‐malonyl)‐glucoside) were highly correlated with the levels of anthocyanin, suggesting that high production of anthocyanin generates more of the shared intermediate, dihydroquercetin, and diverts it to produce flavonol through expression of *FLS*. From our results of the transcriptome analysis, we consider that it is possibly mediated by the action of *MYB12* in lettuce. We found the lettuce *MYB12* homolog among the ABHAG, genes that were highly associated with anthocyanin biosynthesis (Figure S5). *MYB12* has been identified as a flavonol‐specific regulator of phenylpropanoid biosynthesis in *Arabidopsis* and *FLS* is a direct target of MYB12 in *Arabidopsis* (Mehrtens et al., 2005). Coordinated transcriptional regulation of genes involved in branch pathways associated with the anthocyanin biosynthetic pathway would confer a balanced high production of anthocyanin and other phenolic compounds, including flavonoids, in red leaf lettuce cultivars. If the specific MYB transcription factors were solely expressed (Blanco et al., 2018; Liu et al., 2018; Pandey et al., 2015), phenolic compounds might be synthesized independently of anthocyanin biosynthesis. We suggest that, by metabolic engineering for phenolic compounds, which are early by‐products of the phenylpropanoid biosynthetic pathway, it might be possible to give green leaf lettuce nutritionally valuable components other than anthocyanin.

Although we have confirmed that evolutionally conserved components play a critical role in anthocyanin accumulation in lettuce, it is also necessary to optimize the quality and quantity of light to maximize the efficiency of anthocyanin synthesis in closed‐type plant factories equipped with artificial light. It might be possible to find a light environment that can promote efficient accumulation of anthocyanins by using ABHAG as an indicator. Artificial environments are easily controllable and highly reproducible. In addition, the development of light‐emitting diode light sources is progressing steadily. Searching for environments optimized for anthocyanin accumulation in various red lettuce cultivars might help identify novel genetic loci controlling the production of phenolic compounds, including anthocyanin, and will support data‐driven strategies for breeding new cultivars appropriate to cultivation in plant factories.

#### AUTHOR CONTRIBUTION

K.C.W., J.Y., H.I. designed the project and experiments. K.C.W., N.I., Y.N., Z.F., H.I. performed the experiments. K.C.W., H.S., H.Y., H.I. analyzed the data. All authors read and contributed to the editing of the final manuscript.

#### CONFLICTS OF INTEREST

The authors have no conflict of interest to declare.

## Supporting information


Figure S1
Figure S2.
Figure S3.
Figure S4.
Figure S5.
Table S1.

Table S2

## Data Availability

The RNA‐seq data and genome sequencing data that support the major findings of this study are openly available in DDBJ database. Those were deposited in DDBJ repository under accession number DRR374254‐DRR374279 (RNA‐seq) and DRR356564‐DRR356581 (genome sequencing data), respectively.
