# Supplementary material for: Genetic effects of Red Lettuce Leaf genes on red coloration in leaf lettuce under artificial lighting conditions
Source: Plant Environ Interact. 2022 Sep 2;3(4):179–92. doi: 10.1002/pei3.10089 (PMC10168059; doi:10.1002/pei3.10089)
Supplement: Supplementary file 1 — Figure S1 Figure S2. Figure S3. Figure S4. Figure S5. Table S1. [file PEI3-3-179-s001.docx]

**Supporting Information**

**Overview**

**Figures**

- Supporting Information Figure S1. Schematic presentation of the structure of *RLL1* (A), *RLL2* (B), *RLL3* (C), *RLL4* (D), *ANS* (E), and *GST* (F) genes and each nucleotide polymorphism
- Supporting Information Figure S2. Functional *RLL2* copy in the duplicated *RLL2* genes in red leaf cultivars
- Supporting Information Figure S3. *RLL* genotype in 88 leaf lettuce cultivars from around the world
- Supporting Information Figure S4. Abundance of caffeoyltartaric acid and caffeoylmalic acid in nine leaf lettuce cultivars
- Supporting Information Figure S5. Genes that were screened for high correlation with anthocyanin accumulation from RNA-seq data

**Tables**

- Supporting Information Table S1. Multistep solvent gradient program for HPLC separation of flavonoids
- Supporting Information Table S2. Primers used in this study (Excel file)

**Supporting Information Figure S1. Schematic presentation of the structure of *RLL1* (A), *RLL2* (B), *RLL3* (C), *RLL4* (D), *ANS* (E), and *GST* (F) genes and each nucleotide polymorphism**

The boxes indicate exons. The functional nucleotide polymorphisms in individual genes are indicated by red color. Each genotype has a strong (Type A) and weak or no (Type B) effects on anthocyanin accumulation. In *RLL2*, we distinguished a single-copy gene of *RLL2B-Y37* observed in the *L. sativa* cv. Salinas genome and the duplicated region of the *RLL2* locus for genotyping.

**Supporting Information Figure S2. Functional *RLL2* copy in the duplicated *RLL2* genes in red leaf cultivars**

Reads from RNA-seq data were mapped to a contig harboring the duplicated *RLL2* genes constructed from Banchu-Red-Fire genome data and were visualized by IGV (Robinson et al. 2011). GL: Green-Leaf, RO: Red-Oak, RL: Red-Leaf.

**Supporting Information Figure S3.** ***RLL* genotype in 88 leaf lettuce cultivars from around the world**

Genotyping was performed using sequence variations detected in NGS data. Gray and white indicate each genotype with strong (A) and weak or no (B) effects on anthocyanin accumulation, respectively. For *RLL2*, because data for the in/del of 15-bp used for distinguishing the genotype were missing, surrounding polymorphisms were also used as indexes for distinguishing the genotypes.

*1, The description is available on the website of the Open Source Seed Initiative. Retrieved February 1, 2022, from https://osseeds.org/ossi-varieties/flashy-trout-back.

*2, The passport data deposited in the USDA-ARS germplasm database. Retrieved February 1, 2022, from https://npgsweb.ars-grin.gov/gringlobal/accessiondetail?id=1431633.

**Supporting Information Figure S4. Abundance of caffeoyltartaric acid and caffeoylmalic acid in nine leaf lettuce cultivars**

Data are means ± SE (n = 3). Vertical axis shows the signal output of HPLC measured at 350 nm (mAU/g^−1^ fw).

**Supporting Information Figure S5. Genes that were screened for high correlation with anthocyanin accumulation from RNA-seq data**

Each colored box shows the average log_2_ (TPM + 1) value of a metabolic pathway gene, according to the color scale. Asterisks indicate genes shown in Figure 6.

**Supporting Information Table S1. Multistep solvent gradient program for HPLC separation of flavonoids**

**Supporting Information Table S2. Primers used in this study**

(Excel file)
